# Predictive factors for determining first-attempt success on the American Board of Cardiovascular Perfusion Certification Exams for graduates of a master’s level perfusion education program

**DOI:** 10.1051/ject/2025062

**Published:** 2026-03-13

**Authors:** Amber M. Palmer

**Affiliations:** 1 University of Pittsburgh Medical Center Presbyterian Pittsburgh Pennsylvania USA

**Keywords:** Board certification, First-time pass rate, Perfusion coursework, American Board of Cardiovascular Perfusion (ABCP) Exam

## Abstract

*Background:* As demand for perfusionists grows due to increased cardiac procedures and retirements, improving first-time pass rates is essential to addressing workforce shortages. This quantitative study analyzed academic, demographic, and clinical variables as predictors of success on the American Board of Cardiovascular Perfusion (ABCP) exam. *Materials and Methods:* Data were collected from 103 students enrolled in the master’s-level Carlow University–University of Pittsburgh Medical Center (UPMC) Cardiovascular Perfusion program between 2017 and 2022 (IRB #02232024-1). Student-level variables included undergraduate GPA, grades in prerequisite courses, clinical experience hours, and admission status (graduate vs. undergraduate). Associations between these variables and first-time ABCP exam success were examined. *Results:* Logistic regression analysis revealed that higher performance in Introduction to Cardiac Perfusion (*B* = 1.002, *p* = .008) and Hematology (*B* = .636, *p* = .028) significantly predicted first-time success on the ABCP exam. Admission as an undergraduate student was also a significant predictor (*p* = .004). However, neither the number of clinical experience hours nor the student’s cohort year showed statistical significance (*p* > .05). *Conclusion:* Academic performance in key foundational courses and student background (such as admission status) were significantly associated with first-time ABCP exam success. In contrast, the amount of clinical experience did not demonstrate a meaningful impact on pass rates. Further research should utilize a richer dataset that captures a more comprehensive view of perfusion program curricula, including detailed clinical training components. This would help clarify how specific educational experiences contribute to ABCP exam success and overall program outcomes.

## Introduction

While the volume of cardiac cases involving perfusionists continues to increase, the need to replace the aging workforce is imperative due to the retirement of the baby boomer population [1]. Due to the change in the number of providers, perfusionists are in high demand, creating a competitive market for recruiting reliable candidates. Nearly 44% of certified perfusionists are over the age of 50, leading to an annual net loss of about 100 clinicians as retirements outpace new graduates. High vacancy and turnover rates worsen the shortage, highlighting the urgent need for effective recruitment and retention efforts [2]. While the perfusion workforce is continuously progressing, the current needs and changes have not been studied in depth regarding the aging workforce, staffing demands, educational and certification requirements, and advancements in extracorporeal technology [3]. With a limited number of available seats in perfusion programs, administrators strive to investigate academic and non-academic variables that best predict the success of a student in terms of program completion and board certification achievement [4]. Over the last decade, perfusionists’ roles have expanded beyond heart surgery to include managing ECMO in critical care, supporting organ preservation with technologies like “organs in a box” and normothermic regional perfusion (NRP), and assisting with mechanical circulatory devices. While these growing responsibilities have opened up new opportunities, they have also highlighted the urgent need for strategic efforts to address workforce shortages and maintain high-quality patient care [5]. Each year, approximately 140 to 150 students graduate from perfusion programs in the United States. However, 200 to 250 new professionals enter the field annually, as some individuals take their certification exams later. Around 300 perfusionists leave the workforce each year, primarily due to retirement. Early-career attrition is relatively low, with fewer than 5 to 10 percent of new graduates leaving within the first few years. Despite the growing need for perfusionists, expanding program enrollment remains difficult due to limited clinical training sites and strict accreditation requirements that ensure educational quality and patient safety [6]. The review of preadmission variables for entry into the healthcare system is critical to best predict candidates with potential career success [7]. Due to the national shortage of perfusionists, the current demand for successful outcomes has left administrators with the challenge of selecting applicants that exemplify good academic standing based on criteria such as Grade Point Average (GPA), standardized testing, interviews, and coursework [5]. While it is difficult to select one variable that predicts the success of a candidate, decisions must be made based on data to limit the number of unqualified applicants and reduce attrition [7].

The purpose of this quantitative study was to provide a preliminary analysis of variables including academic courses, demographics, and prior clinical experience used as predictive measures for success on the ABCP examination. The ABCP, established in 1975, took over certification from The American Society of Extracorporeal Technology (AmSECT) and later limited exam eligibility to graduates of accredited perfusion programs. Certification requires passing two parts, the Perfusion Basic Science Examination (PBSE) and the Clinical Applications in Perfusion Examination (CAPE). The PBSE exam focuses on perfusion science and extracorporeal support, and the CAPE exam focuses on clinical scenarios. Both exams are offered twice a year, in the spring and fall. Candidates are eligible to take the PBSE after completing the didactic portion of an accredited program and may take the CAPE after meeting the required number of clinical cases. As a result, it is possible to take the PBSE without yet qualifying for the CAPE. However, it is important to highlight that the analysis was limited to first-time pass rates and did not consider candidates who passed one section of the exam but failed another, thus excluding partial exam outcomes from the evaluation.

The UPMC School of Cardiovascular Perfusion, in partnership with Carlow University, is a five-year dual-degree pathway that combines a Bachelor of Science in Biology with a Master of Science in Cardiovascular Perfusion. Select students complete foundational science courses during their undergraduate years at Carlow University, then transition into graduate-level coursework and hands-on clinical training at UPMC. Students who complete the undergraduate portion at Carlow earn their bachelor’s degree within the first four years and complete the 18-month master’s program alongside the graduate students. The graduate-level coursework in the Carlow–UPMC Perfusion Program is closely aligned with the content of the ABCP examination topics. Each year, a perfusion class of approximately 20 students is selected from a combination of Carlow University undergraduates and about 100 external applicants with a bachelor’s degree from other institutions. Admission is based on a science GPA of 3.5 or higher, relevant medical or healthcare experience, interviews, and letters of recommendation. Carlow undergraduates who meet the program’s admission requirements are given priority consideration, and the number of external applicants accepted depends on how many Carlow students qualify for admission.

## Methods and materials

This study examined retrospective data related to the preadmission variables of Carlow University–UPMC perfusion program students from 2017 to 2022 and included 103 students. For this study, student-level data included variables such as undergraduate GPA, grades in prerequisite science courses, amount of clinical experience prior to admission, and admission status (undergraduate vs. external applicant). Success on the ABCP exam was identified to evaluate which variables predicted first-time pass rates. IRB approval from Carlow University was granted for the use of student-level data.

In combination with the literature review on academic and non-academic factors that affect success on the ABCP exam, the following research questions were explored:

1. What courses affect the ABCP certification exam pass/fail outcome during undergraduate and perfusion school?
2. What applicant factors, such as demographics, degree type, and prior medical experience, affect pass/fail score on the ABCP certification exam?
3. Does more clinical experience, including rotation site (e.g., outside of UPMC) or cohort year, affect the ABCP certification exam pass/fail score?

The dependent variable was the student’s ABCP exam outcome, defined as a pass or fail on both components of the certification exam and the independent variables included grades from select undergraduate Carlow University courses, UPMC perfusion program courses, and applicant factors such as education background, gender, and cohort. The research questions were selected based on data available through the Carlow University–UPMC Perfusion Program. Table 1 summarizes the variables that correspond to each research question. Although the information was specific to this program, the research questions were designed to be applicable to other perfusion education programs as well. Personal information was deidentified to ensure confidentiality. The academic advisor ensured data accuracy, added ABCP results, and recorded additional applicant factors. Data was analyzed using IBM Statistical Package for the Social Sciences (SPSS) software, and the coded data was securely stored on a password-protected UPMC computer, with the aim of providing insights into which preadmission variables best predict first-time pass rates on the ABCP exam.

**Table 1:** General description of variables.

Variable	*n* (%) or mean ± SD
Cohorts included:	2017–2022
Total students:	103
Gender	
Male	39 (37.9%)
Female	64 (62.1%)
ABCP exam outcomes:	
Passed on first attempt	77 (74.8%)
Failed on first attempt	26 (25.2%)
Student type:	
Carlow undergraduate	85 (82.5%)
Graduate student	13 (12.6%)
Courses included in analysis:	
Intro to cardiac perfusion	Percentile group
Intro to cardiovascular surgery	Percentile group
Congenital pathology	Percentile group
Renal anatomy and physiology	Percentile group
Cardiovascular pharmacology	Percentile group
Acquired pathology	Percentile group
Pulmonary anatomy and physiology	Percentile group

A regression analysis allowed academic and non-academic variables to be studied against the performance on the ABCP exam. The research plan consisted of binomial logistic regression analysis, which aimed to predict the probability of a dichotomous dependent variable based on multiple independent variables. The Hosmer–Lemeshow test was performed to evaluate how well the predicted probabilities of the logistic regression model matched the actual outcomes of the data observed for research question one. This test compares the predicted probabilities from the regression model to the actual outcomes, and a high p-value (*p* > .05) indicates a good fit, meaning there is no significant difference between expected and observed results.

During model development for Research Question One, the Hosmer–Lemeshow test was used as a diagnostic tool to determine which academic variables – specifically course grades – contributed to a well-fitting model. Together, the binary logistic regression model and the Hosmer–Lemeshow test provided a comprehensive approach to modeling and evaluating the binary outcomes in statistical analysis. A high *p*-value (>.05) suggested the model fits the data well, meaning there were no significant differences between the observed and expected outcomes among the variables. For this model, the p-value = .164.

Because the Hosmer–Lemeshow test had to show the model was a good fit, additional undergraduate and graduate courses were not included in the test due to a low *p*-value (<.05) indicating a poor fit of the model. This suggested that the predicted probabilities based on these variables did not accurately match the observed ABCP exam outcomes. Several attempts were made to use as many of the undergraduate and perfusion classes as possible. However, there was skewed data which impacted the overall fit of the regression models. The Hosmer–Lemeshow test thus helped identify which courses could be retained without compromising model fit. Based on these factors, all undergraduate courses were left out of the regression. These courses included anatomy and physiology, calculus, ecology, organic chemistry, medical physics, etc.

For research questions two and three, binary variables were created to convert categorical values such as “Male” and “Female” into numerical form, with “male” coded as 1 and “female” as 0. To avoid multicollinearity in the regression model, one fewer binary variable than the number of categories was used. The omitted category serves as the reference group for comparison. In cases with two categories, one category is designated as the reference code. For research question two, the demographic variable included “male” and “female.” The education variable categorized students as “undergrad” if they attended Carlow University prior to entering the perfusion program, and “transfer” if they did not. For the experience variable, “yes” indicated students with prior medical experience, while “no” indicated those without it. For the demographic variable, “male” was used as the reference code and “female” was the dummy variable. For the education variable, “transfer” was used as the reference code and “undergraduate” was used as the dummy variable. For the experiential variable, “yes” was used as the reference code and “no” was used as binary variable.

## Results

To evaluate the first research question, what courses affect the ABCP certification exam pass/fail outcome during graduate (perfusion) school, the logistic analysis in Table 2 shows the quintiles created for each course included in the regression. To improve the model’s performance and interpretability, course grades were transformed into quintiles, allowing for more balanced groupings and reducing skewed data. This transformation allowed for a better-fitting model (*p* > .05) while preserving meaningful insights into the relationship between academic performance and exam outcomes. Each variable in the equation is represented by the percentile group of each graduate school class. Because the model was not of sound fit (*p* < .05), the undergraduate courses were left out of the regression. Students in the higher quintile were more likely to pass the exam on the first attempt (*p* < .05). The unstandardized B (beta) = 1.002 indicated students in the higher percentile group of Intro to Cardiac Perfusion were more likely to pass the exam on the first attempt, *p* = .008. The unstandardized B (beta) = .636 indicated students in the higher percentile group of Hematology were more likely to pass the exam on the first attempt, *p* = .028. The percentile group of Pulmonary Anatomy and Physiology was starting to reach statistical significance, with *p* = .089. The remaining course variables such as Intro to Cardiovascular Surgery, Congenital Pathology, and Cardiovascular Pharmacology were not statistically significantly related to the dependent variable pass or fail the ABCP examination (*p* > .05).

**Table 2:** Research question one: Graduate perfusion courses included in the regression.

Variables in the equation	*B*	Sig.
Percentile group of intro to cardiac perfusion	1.002	.008
Percentile group of intro to cardiovascular surgery	.015	.963
Percentile group of cardiovascular physiology	.083	.792
Percentile group of congenital pathology	.016	.959
Percentile group of hematology	.636	.028
Percentile group of renal anatomy and physiology	−.001	.999
Percentile group of cardiovascular pharmacology	.029	.926
Percentile group of acquired pathology	.180	.540
Percentile group of pulmonary anatomy and physiology	−.620	.089

To answer the second research question, what applicant factors (demographic, educational, and experiential) affect pass/fail outcome on the ABCP certification exam, the logistic regression analysis in Table 3 shows the binary variables created for each of the applicant factors included in the regression. The regression identified an independent applicant variable, admission: undergraduate, which statistically significant (*p* = .004) indicating students were more likely to pass the exam on the first attempt. The independent applicant variable, experience: no, was starting to reach statistical significance, because *p* = .097.

**Table 3:** Research question two: Applicant factors included in the regression.

Variables in the equation	*B*	Sig.
Admission: undergrad	2.362	.004
Experience: no	−1.719	.097
Gender: female	.395	.594

The third research question sought to determine the impact of clinical experience, such as rotation site or cohort, on the ABCP certification exam pass/fail score; the logistic regression analysis in Table 4 shows the dummy variables created for each cohort from 2017 to 2021 for inclusion in the regression analysis, with the 2022 cohort used as the reference group. The regression showed that the independent variable, more clinical experience or cohort, was not statistically significantly related to the dependent variable, pass/fail the ABCP examination (*p* > .05).

**Table 4:** Research question three: Cohorts included in the regression.

Variables in the equation		*B*	Sig.
Step 1^a^	Cohort class of 2017 and 2018	−1.563	.227
	Cohort class of 2019	−1.037	.390
	Cohort class of 2020	−.912	.429
	Cohort class of 2021	−.375	.756

## Discussion

The results of this study offer a foundational reference point for evaluating and potentially enhancing the perfusion program’s admissions process and educational standards. However, due to limitations in the data provided by the registrar, this analysis was restricted to coursework completed within the UPMC Perfusion Program, excluding academic performance from Carlow University undergraduate courses. As a result, the scope of the study was narrowed, and only a select number of variables could be analyzed. The reliance on existing institutional data limited the ability to investigate broader pre-admission indicators, such as cumulative undergraduate GPA, prerequisite science course performance, standardized test scores, healthcare experience, or personal attributes like resilience and communication skills. To conduct more comprehensive and predictive research on what defines a successful perfusion student, future studies would require access to a more complete dataset that includes both academic and non-academic pre-admission variables. Collecting and analyzing this expanded data would allow for the development of a strong candidate profile and could inform data-driven adjustments to admissions criteria aimed at optimizing student success and certification outcomes. If other perfusion education programs were to compile similarly detailed datasets from their previous classes, this research framework could be applied more broadly, enabling comparative analyses across institutions.

Research question one addressed the courses that affect the ABCP certification exam pass/fail outcome. This analysis found that students who were in the percentile group of Intro to Cardiac Perfusion and the percentile group of Hematology were more likely to pass the ABCP exam on the first attempt. The groups were broken up into quintiles and students in the upper quintile were statistically more likely to pass the exam. The percentile group of Pulmonary Anatomy and Physiology was close to significance but *p* > .05. The remaining courses did not show significance in the regression. While only two courses showed significance, the study supported the literature in regard to coursework predicting successful outcomes. This study focused on perfusion courses due to discrepancies in the dataset. Of the courses that showed significance, the percentile of Intro to Cardiac Perfusion was a noteworthy finding suggesting that students who start off the perfusion program in the higher quintile may show promising outcomes during the first attempt on the ABCP examination. The percentile of Hematology was an interesting finding, as it focuses more on blood components than on complex physiological systems typically emphasized on the ABCP exam. Its significance may indicate that strong performance in foundational science or solid study habits correlates with overall exam success.

Research question two addressed applicant factors that affect the ABCP certification exam pass/fail outcome. This study found that students who were in the variable “undergraduates” from Carlow University were more likely to pass the ABCP exam on the first attempt. The group of students with no prior experience was close to significance but *p* > .05. The remaining variable “female” did not show significance in the regression. The variable representing traditional Carlow undergraduate students was a notable finding, indicating that those who transition directly from Carlow’s undergraduate program into the perfusion program are more likely to pass the ABCP exam on their first attempt. This outcome was anticipated by the researcher, as students following the traditional route may benefit from a more consistent and cohesive educational experience. The variable of students having “no experience” was an unexpected finding to the researcher because even though *p* = .097, the regression implied further research could be done to identify if prior work experience indicates an increase in first-time pass rate on the ABCP exam.

Research question three addressed how clinical experience affects the ABCP certification exam pass/fail outcome. This study found that students who were in any of the variables for cohorts showed no significance in passing the ABCP exam on the first attempt. Although the 2020 cohort experienced reduced clinical exposure due to COVID-19 restrictions, this did not appear to impact their pass rates. Despite initial expectations that limited hands-on experience might affect performance, the pandemic did not influence students’ success on the ABCP examination.

The results of this study provide insight for Carlow–UPMC administration regarding undergraduate success rate on the ABCP exam. The data suggests that admitting a larger number of undergraduate students to perfusion school rather than increasing the number of graduate students shows better success on the exam. The current perfusion admission demographics are undergraduate students and few graduate students. Rather than make the demographic more even, Carlow stakeholders could argue that undergraduate students have a higher first attempt pass rate on the ABCP exam.

## Conclusion

A key recommendation for future research is to involve multiple perfusion programs to increase sample size and diversity. A larger dataset would enable more robust analyses of both undergraduate and graduate coursework, enhancing the depth and generalizability of findings. Additionally, documenting transfer student grades within university databases would improve data reliability for future studies. Research should also explore innovative teaching methods and clinical training models to better prepare students for professional practice. Extended follow-up studies tracking graduates’ clinical performance are essential to understand how educational experiences influence long-term career success. Expanding research in these areas will support improvements in admissions, curriculum design, and clinical training, ultimately raising the overall quality of perfusion education. Broadening research efforts across institutions will not only improve individual student outcomes but also strengthen the profession’s credibility and visibility within healthcare. These advancements can further improve patient outcomes, strengthen interdisciplinary collaboration, and reinforce the essential role of perfusionists within cardiovascular care teams.

## Limitations

A key limitation of this case study was its limited generalizability due to a small sample size, which restricted the statistical power to detect significant effects. Several undergraduate courses were excluded from analysis because of skewed data and poor model fit, reducing the ability to assess their impact on ABCP exam success and possibly overlooking important predictors. Additionally, the lack of access to broader pre-admission variables made it difficult to explore causation or control for external factors influencing outcomes.

## Data Availability

All available data are incorporated into the article.
